# Influence of collateral circulation on cerebral blood flow and frontal lobe cognitive function in patients with severe internal carotid artery stenosis

**DOI:** 10.1186/s12883-019-1380-9

**Published:** 2019-07-05

**Authors:** Wei Wei, Xingyang Yi, Jianghai Ruan, Xiaodong Duan, Hua Luo, Zhiyu Lv

**Affiliations:** 1 Department of Neurology, the Affiliated Hospital of Southwest Medical University, No. 25 Taiping Road, Luzhou, 646000 Sichuan China; 2 Department of Neurology, People’s Hospital of Deyang City, Deyang, 618000 Sichuan China; 3 Department of Rehabilitation medicine, the Affiliated Hospital of Southwest Medical University, Luzhou, 646000 Sichuan China

**Keywords:** Internal carotid artery, Stenosis, Collateral circulation, Cerebral blood flow, Cognitive function

## Abstract

**Background:**

This study aimed to investigate the cerebral blood flow (CBF) and frontal lobe cognitive function in severe internal carotid artery (ICA) stenosis patients with different types of collateral circulation.

**Methods:**

One hundred twenty-six patients with severe unilateral ICA stenosis were enrolled. Digital subtraction angiography (DSA) was performed to recruit patients with one of three common types of collateral circulation: anterior communicating artery (AcoA), posterior communicating artery (PcoA) and ophthalmic artery (OA). The hemodynamic parameters of the middle cerebral artery (MCA) were measured using transcranial Doppler (TCD), and the individual frontal lobe cognitive attention functions were evaluated using Word Fluency Test, Trail-Making Test (TMT), Digit Span, and Stroop Color Word Test (SCWT). The correlation between hemodynamic changes and the scores of all tasks was analyzed.

**Results:**

On the side of arterial stenosis, the CBF velocities were highest in AcoA group and lowest in the OA group. All patients performed worse in TMT and Digit Span than the matched normal controls. The AcoA group exhibited a lower pulsatility index (PI) and a longer response time in the Stroop task, but had a higher accuracy rate in the Stroop task and higher scores in Word Fluency Test than the PcoA and OA groups. In all the three groups, PI was positively correlated with the accuracy rate for Stroop interference effects.

**Conclusions:**

Our findings suggested that the frontal lobe cognitive function of patients with ICA was impaired, and AcoA collaterals may be beneficial for selective attention functions, whereas OA collaterals may be associated with impairment of selective attention functions. Additionally, a high PI may be an indicator for identifying impaired selective attention in patients with severe ICA stenosis.

## Background

Severe internal carotid artery (ICA) stenosis is characterized by chronic cerebral hypoperfusion due to the decreased cerebral blood flow (CBF). It has been proven that long-lasting cerebral hypoperfusion may impair energy metabolism in neurons and lead to cognitive impairment [1, 2]. Although cerebral hypoperfusion is common in patients with ICA stenosis, collateral circulation provides new paths to maintain sufficient blood supply even though it may only represent a poor compensation [2]. Some studies have investigated the association between collateral circulation and cognitive dysfunction in recent years; however, the findings on this topic have been inconsistent [3, 4]. Everts et al. found no correlation between the formation of collaterals and cognitive function [5], while Sztriha et al. proposed that collaterals-related perfusion restoration could improve the cognitive dysfunction [6]. Thus far, there has not yet been a study investigating the specific association between different types of collateral circulation and cerebral hemodynamics as well as cognitive function in patients with severe ICA stenosis.

Word Fluency Test is a sensitive measure for frontal lobe cognitive functions [7], and Trail-Making Test (TMT) is frequently used in neuropsychological assessments to estimate executive function [8]. Forward and Reverse Digit Span tests evaluate working memory. Stroop Color Word Test (SCWT) is a classic, widely-used psychological evaluation tool that ignores irrelevant information and enhances the ability of suppressing interference which delays the response [9]. It demands resolution of a conflict reading and naming, which require selective attention [10]. Furthermore, selective attention refers to a top-down information processing to selectively filter information, which can be adjusted by enhancement or inhibition of neural activities associated with pulvino-cortical networks [11] and dorsolateral prefrontal cortex [12]. Collectively, the aforementioned tests call for the function of frontal lobe, which could be influenced by the blood supply of ICA.

Thus, we hypothesized that patients with severe ICA stenosis may show different behavioral performance in frontal lobe cognitive functions due to diverse collateral compensations. The present study aimed to investigate the cerebral blood flow (CBF) and frontal lobe cognitive function in severe ICA stenosis patients with different types of collateral circulation.

## Methods

### Participants

This study was approved by the Ethics Committee of the Affiliated Hospital of Southwest Medical University. Written informed consent was obtained from each participant.

A total of 126 patients with severe unilateral ICA stenosis were enrolled between January 2013 and October 2017. The diagnosis of ICA stenosis was confirmed by digital subtraction angiography (DSA) (stenosis ≥70%) according to the North American Symptomatic Carotid Endarterectomy Trail (NASCET) criteria [13]. Patients with a single type of collateral circulation: anterior communicating artery (AcoA), posterior communicating artery (PcoA), or ophthalmic artery (OA) confirmed by DSA were selected. The exclusion criteria included: 1) dementia (Montreal Cognitive Assessment < 26) [14]; 2) disturbance of consciousness; 3) transient ischemic attack (TIA); 4) MRI showing cerebral infarction or other pathological brain diseases, such as hemorrhages, tumors, aneurysms and vascular anomalies; 5) inadequate temporal window in TCD examination; 6) concomitant stenosis in other arteries; or 7) color blindness or color weakness. According to the type of collateral circulation, patients were classified into three groups: AcoA group, PcoA group, and OA group. Eighty-one control participants matched for age, sex, and education, were enrolled from the individuals who underwent DSA with normal results. The exclusion criteria were identical to those applied for ICA stenosis patients.

### TCD examination

Blood flow velocity (BFV) of the ipsilateral MCA was measured at a depth of 50 mm by TCD, placing a handheld transducer over the temporal bone, using a 2-MHz TCD device (EMS-9A, Delica, China). The BFV parameters included systolic flow velocity (Vs), diastolic flow velocity (Vd) and mean flow velocity (Vm). The pulsatility index (PI) was calculated as follows: PI = (Vs - Vd) / Vm.

### Neuropsychological evaluation

The Word Fluency Test required participants to give vegetable words within 1 min [15]. TMT was composed by Part A and Part B [8]. Part A requires participants to draw a line connecting 25 encircled number distributed in a piece of paper consecutively; Part B requires participants to connect the alternating encircled numbers and letters consecutively. It was rated by the time used in finishing each part. Digit Span required participants to repeat a series of increased length of numbers in forward and reverse order [16].

Selective attention was evaluated using SCWT. Four color names in Chinese (red, yellow, green, and blue) were presented on the computer screen. First, the name of one color was presented in black, and the subject was instructed to respond by pressing the corresponding key (SCWT A). Second, patches colored in one of these colors were presented, which was judged by the subject (SCWT B). Third, the color of the word did not match the meaning of the word, and the subject was requested to judge the color ignoring the incongruent meaning (SCWT C). Each subtask contains 50 stimuli and should be completed as fast as possible. The response time (RT) and accuracy rate (AR) were recorded. RT for Stroop interference effects (SIE) was calculated as “RT_SCWT C_ - RT_SCWT B_” . The AR for SIE was calculated as “AR_SCWT B_- AR_SCWT C_” [17]. Each subject was provided the explanation of SCWT, and practiced the paradigm a trial of 18 rounds (six rounds per subtask).

### Statistical analysis

All statistical analyses were performed using SPSS software (Version 24.0, SPSS Inc., Chicago, IL, USA). Continuous variables were expressed in median and interquartile values. Chi-squared test was used for the analyses of categorical variables, and non-parametric Kruskal-Wallis and post hoc analysis were applied for continuous variables. Correlation between the BFV parameters and neuropsychological scores were analyzed using Spearman’s correlation analysis. Probability (*P*) values ≤0.05 were considered statistically significant.

## Result

### Clinical characteristics

Among the 126 patients with severe unilateral ICA stenosis, 105 patients and all the normal controls completed all the examinations and tests. Based on their results, 65 patients were assigned to the AcoA group, 28 patients to the PcoA group, and 12 patients to the OA group. Demographic data and risk factors were summarized (Table 1). There was no significant difference in these demographic characteristics and risk factors among the four groups (all *P* > 0.05). In the AcoA group, the incidence of diabetes mellitus (*χ*^*2*^ = 4.125, *P* = 0.042) and hyperlipidemia (*χ*^*2*^ = 5.395, *P* = 0.02) were slightly higher than those in controls after pairwise comparisons.

**Table 1:** Demographic data of patients with severe ICA stenosis and normal controls

Characteristics	AcoA group	PcoA group	OA group	Controls	H or *χ*^*2*^	*P* value
Number	65	28	12	81		
Age (years)	68.00 (60.50,72.00)	67.50 (62.25,71.75)	62.00 (59.00,68.25)	69.00 (62.00,72.00)	6.684	0.083
Sex (M/F)	47/18	18/10	8/4	53/28	1.420	0.701
Education (years)	8.00 (5.00,12.00)	8.50 (5.00,12.00)	7.50 (3.75,10.75)	6.00 (3.50,10.00)	3.158	0.368
Hypertension	48 (73.8%)	19 (67.9%)	8 (66.7%)	48 (59.3%)	3.474	0.324
Diabetes mellitus	35 (53.8%)	12 (42.9%)	6 (50%)	30 (37.0%)	4.300	0.231
Hyperlipidemia	51 (78.5%)	20 (71.4%)	9 (75%)	49 (60.5%)	5.765	0.124
Smoking history	42 (64.6%)	19 (67.9%)	7 (58.3%)	50 (61.7%)	0.512	0.916
Alcoholism	10 (15.4%)	5 (17.9%)	2 (16.7%)	20 (24.7%)	2.151	0.542

*χ*^*2*^ value, by Chi-squared test. *H* and *P* value, by non-parametric Kruscal-Wallis tests within the four groups

### Influence of collateral circulation on BFV

The ipsilateral BFV parameters (Vs, Vm, and Vd) in patients with ICA stenosis were significantly lower than those in normal controls (*P* < 0.0001). These BFV parameters from high to low in order were AcoA group > PcoA group > OA group (all *P* < 0.05). PI in patients was significantly lower than that in normal controls, and PI from high to low in order was OA group > PcoA group > AcoA group (all *P* < 0.05). The statistical results were presented in Table 2. The contralateral BFV parameters (Vs, Vm, and Vd) only in the AcoA group were significantly higher than those in the other groups (all *P* < 0.0001). There was no significant difference in contralateral PI among the groups (*P* > 0.05) (Table 3).

**Table 2:** The *P* values of ipsilateral BFV parameters between different groups

	Vs	Vm	Vd	PI
AcoA vs PcoA	0.017	< 0.0001	< 0.0001	0.034
AcoA vs OA	< 0.0001	< 0.0001	< 0.0001	0.032
AcoA vs controls	< 0.0001	< 0.0001	0.036	< 0.0001
PcoA vs OA	0.003	0.002	0.007	0.997
PcoA vs controls	< 0.0001	< 0.0001	< 0.0001	< 0.0001
OA vs controls	< 0.0001	< 0.0001	< 0.0001	< 0.0001

Vs, stolic flow velocity; Vm, mean flow velocity; Vd, diastolic velocity; PI, pulsatility index. *P* value, after post Hoc tests

**Table 3:** TCD parameters in patients with severe ICA stenosis and normal controls

Parameters	AcoA group	PcoA group	OA group	Controls	H	*P* value
Vs (Affected side)	50.00 (44.50,56.00)	44.00 (36.25,52.25)	31.50 (27.25,41.25)	79.00 (74.00,85.00)	143.610	< 0.0001^*^
Vs (Unaffected side)	123.00 (102.00,136.50)	80.50 (68.25,96.00)	96.00 (69.00,107.25)		102.901	< 0.0001^*^
Vm (Affected side)	39.00 (35.00,43.50)	32.50 (28.25,38.00)	22.50 (20.25,30.50)	52.00 (46.00,57.00)	117.469	< 0.0001^*^
Vm (Unaffected side)	73.00 (63.00,86.50)	47.50 (42.25,61.50)	55.50 (41.25,67.25)		78.395	< 0.0001^*^
Vd (Affected side)	33.00 (28.00,37.00)	28.00 (23.25,31.75)	18.50 (17.00,25.50)	37.00 (32.75,40.00)	55.753	< 0.0001^*^
Vd (Unaffected side)	51.00 (42.00,64.50)	31.50 (28.00,43.75)	35.50 (28.50,44.50)		57.931	< 0.0001^*^
PI (Affected side)	0.45 (0.35,0.54)	0.55 (0.42,0.60)	0.53 (0.50,0.59)	0.85 (0.75,1.03)	131.688	< 0.0001^*^
PI (Unaffected side)	0.88 (0.74,1.11)	0.96 (0.85,1.13)	0.99 (0.87,1.15)		6.445	0.092

Vs, stolic flow velocity; *Vm*, mean flow velocity; *Vd*, diastolic velocity; *PI*, pulsatility index; *H* and *P* value, by non-parametric Kruscal-Wallis tests within the four groups

^*^P < 0.0001 indicate statistically significant differences

### Influence of collateral circulation on neuropsychological tests

Word Fluency Test scores of the AcoA group and controls were significantly higher than those in the PcoA and OA groups (AcoA vs PcoA, *P* = 0.005; AcoA vs OA, *P* = 0.012; PcoA vs controls, *P* < 0.0001; OA vs controls, *P* = 0.001), and there was no significant difference between the AcoA group and controls. All patients required significantly longer time to finish TMT (Part A and Part B) than controls (for Part A: AcoA vs controls, *P* = 0.013; PcoA vs controls: *P* = 0.018; OA vs controls, 0.023; for Part B: AcoA vs controls, *P* < 0.0001; PcoA vs controls, *P* = 0.002; OA vs controls, *P* = 0.001). The scores of Digit Span (forward and reverse) in all patients were significantly lower than those of the control participants (for forward: AcoA vs controls, *P* = 0.01; PcoA vs controls, *P* = 0.009; OA vs controls, *P* = 0.016; for backward: every patient group vs controls, *P* < 0.0001). There was no significant difference among the three patient groups in TMT (Part A and Part B) and Digit Span (*P* > 0.05) (Table 4).

**Table 4:** Comparison of cognitive assessment in patients with severe ICA stenosis and normal controls

	AcoA group	PcoA group	OA group	Controls	H	*P* value
Word Fluency Test	15.00 (13.00,18.50)	13.00 (10.00,15.75)	10.50 (9.00,16.00)	16.00 (14.00,18.50)	25.703	< 0.0001^*^
TMT(s)						
TMT-A	33.51 (28.61,40.23)	37.89 (28.68,41.58)	38.50 (32.22,43.54)	28.82 (25.00,34.63)	17.850	< 0.0001^*^
TMT-B	68.11 (62.06,74.53)	68.26 (60.48,75.52)	73.50 (68.54,78.51)	60.21 (55.36,68.78)	28.739	< 0.0001^*^
Digit Span						
Digit Span forward	6.00 (5.00,7.00)	6.00 (4.25,7.00)	5.00 (4.25,6.75)	7.00 (6.00,8.50)	17.742	< 0.0001^*^
Digit Span backward	3.00 (2.00,4.00)	3.00 (3.00,4.00)	3.50 (3.00,4.00)	5.00 (4.00,6.00)	74.082	< 0.0001^*^

TMT, Trail-Making Test; *H* and *P* value, by non-parametric Kruscal-Wallis tests within the four groups

^*^P < 0.001 indicate statistically significant differences

There was no significant difference in RT_SCWT A_ or RT_SCWT B_ among the four groups (*P* > 0.05). RT_SCWT C_ and RT for SIE in all patient groups were significantly longer than those in normal controls (for SCWTC: AcoA vs controls, *P* < 0.0001; PcoA vs controls, *P* = 0.002; OA vs controls, *P* = 0.043; for RT for SIE: AcoA vs controls, *P* < 0.0001; PcoA vs controls, *P* = 0.021; OA vs controls, *P* = 0.005), and the AcoA group had higher score than the PcoA groups (for SCWT C: *P* = 0.001; for RT for SIE: *P* = 0.02) and OA groups (for SCWT C: *P* = 0.046; for RT for SIE: *P* = 0.03). There was no significant difference in AR_SCWT A_ or AR_SCWT B_ among the four groups (*P* > 0.05). AR_SCWT C_ in the OA group was significantly lower than that in the normal controls (*P* = 0.004), while there was no significant difference in AR_SCWT C_ among AcoA group, PcoA group and normal controls (*P* > 0.05). AR for SIE in PcoA group and OA group was significantly higher than that in AcoA group (for PcoA group, *P* = 0.03; for OA group, *P* = 0.026) and controls (for PcoA group, *P* = 0.001; for OA group, *P* = 0.015), which also caused differences among the four groups (*P* < 0.0001) (Table 5).

**Table 5:** SCWT assessment in patients with severe ICA stenosis and normal controls

SCWT parameters	AcoA group	PcoA group	OA group	Controls	H	*P* value
Response time (ms)						
SCWT A	30.00 (21.50,36.50)	29.00 (20.00,37.50)	28.00 (21.75,34.00)	28.00 (18.50,33.00)	2.209	0.566
SCWT B	49.00 (41.00,61.50)	49.00 (42.25,52.75)	47.50 (42.00,54.25)	46.00 (38.50,52.50)	4.842	0.184
SCWT C	104.00 (94.50,117.50)	96.00 (82.50,106.50)	92.50 (86.75,102.75)	82.00 (70.00,91.00)	65.925	< 0.0001^**^
Response time for SIE	55.00 (48.00,63.00)	52.00 (32.00,57.00)	43.50 (37.75,52.25)	36.00 (27.50,41.00)	70.771	< 0.0001^**^
Accuracy rate (%)						
SCWT A	98.00 (92.50,100.00)	99.00 (96.25,99.75)	97.00 (95.25,99.75)	98.00 (96.00,99.50)	1.134	0.726
SCWT B	85.00 (79.00,96.50)	90.00 (85.00,97.75)	83.50 (78.75,98.25)	89.00 (86.00,95.00)	4.924	0.177
SCWT C	81.00 (75.50,90.00)	81.00 (75.25,90.75)	78.50 (69.75,86.00)	85.00 (80.00,90.00)	11.579	0.009^*^
Accuracy rate for SIE	4.00 (2.50,6.00)	7.00 (5.25,11.75)	9.00 (5.50,11.75)	4.00 (2.00,5.00)	35.621	< 0.0001^**^

*SIE*, Stroop interference effects; *H* and *P* value, by non-parametric Kruscal-Wallis tests within the four groups

^*^P < 0.01, ^**^P < 0.001 indicate statistically significant differences

### Correlation between BFV parameters and neuropsychological scores

**Fig. 1:**
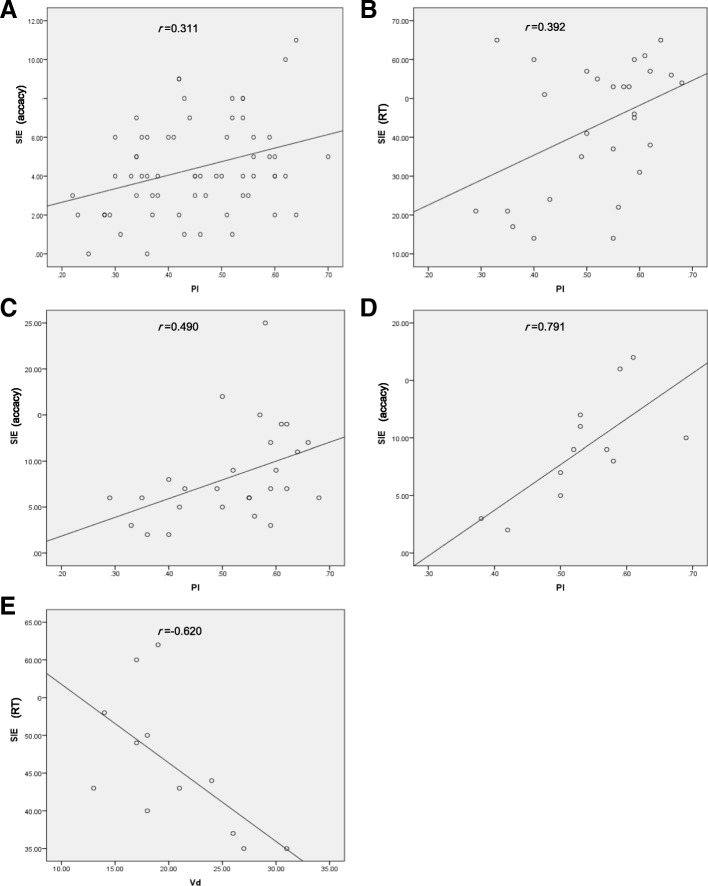
Correlation between TCD parameters (side of stenosis) and SIE scores. **a** Scatter plot demonstrating correlation between pulsatility index (PI) and SIE accuracy in the AcoA group. **b** Scatter plot demonstrating correlation between PI and reaction time of SIE in the PcoA group. **c** Scatter plot demonstrating correlation between PI and SIE accuracy in the PcoA group. **d** Scatter plot demonstrating correlation between PI and SIE accuracy in the OA group. **e** Scatter plot demonstrating correlation between diastolic velocity and reaction time of SIE in the OA group

There was no significant correlation between BFV parameters and Word Fluency Test or Digit Span. The ipsilateral PI was positively correlated with AR for SIE in patients with ICA stenosis (*P* < 0.05), especially in PcoA group and OA group (*P* < 0.01). The ipsilateral PI was positively correlated with RT for SIE in the PcoA group (*P* < 0.05). There was a negative correlation between Vd and RT for SIE in the OA group (*P* < 0.05) (Table 6) (Fig. 1).

**Table 6:** Correlation coefficient between TCD parameters (side of stenosis) and SIE scores

	AcoA group		PcoA group		OA group		Controls	
	RT for SIE	AR for SIE	RT for SIE	AR for SIE	RT for SIE	AR for SIE	RT for SIE	AR for SIE
Vs	0.041	0.172	0.289	−0.030	−0.557	0.214	0.095	−0.007
Vm	0.073	0.071	0.211	−0.149	− 0.566	0.153	0.042	0.023
Vd	0.084	−0.015	0.034	−0.234	−0.620^*^	0.125	0.013	0.096
PI	−0.103	0.311^*^	0.392^*^	0.490^**^	0.143	0.791^**^	0.054	−0.098

*RT*, response time; *AR*, accuracy rate; *SIE*, Stroop interference effects; Vs, systolic flow velocity; *Vm*, mean flow velocity; *Vd*, diastolic flow velocity; *PI*, pulsatility index

^*^P < 0.05, ^**^P < 0.01 indicate statistically significant correlations

## Discussion

In the present study, we investigated whether cerebral BFV and frontal lobe cognitive function would be influenced by the different type of collateral circulation in patients with ICA stenosis. Previous studies have proposed that BFV measured by TCD was a major indicator reflecting cerebral hemodynamics [18], and BFV of MCA is the most reliable parameter [19]; herein, we measured BFV in bilateral MCA.

On the other hand, Word Fluency Test, TMT, Digit Span, and SCWT were also included in the frontal lobe cognitive function assessment [20]. Our findings indicated that SCWT was affected by collaterals more than other neurological function tests. Moreover, we found the ipsilateral PI was significantly associated with impaired selective attention. In brief, the current study provides a new insight into the correlations among collateral circulation, cerebral perfusion and selective attention.

### Relationship between CBFV and collateral circulation

Generally, ICA stenosis mainly affects the blood perfusion of ipsilateral anterior circulation. We found the BFV of ipsilateral MCA was significantly reduced in patients with ICA stenosis. Especially, the decrease of BFV in PcoA group was more apparent than that in AcoA group, suggesting collateral circulation through the AcoA may provide a better compensation for the impaired cerebral perfusion. However, Gfusi et al. proposed that collateral circulation through PcoA to be important for cerebral perfusion because the absence of PcoA collaterals was found to be associated with poor prognosis [21]. Thus, we also speculate collateral circulation through the PcoA may be necessary in the absence of AcoA collaterals. Additionally, the BFV of contralateral MCA in AcoA group was higher than that in other groups, which may be attributed to the compensation.

The effect of OA collaterals on hemispheric hemodynamics in patients with severe ICA stenosis is still controversial. Telman et al. reported that collateral circulation through OA had no influence on hemispheric hemodynamics [22]; nevertheless, Henderson et al. found that OA collaterals may be associated with a lower risk of stroke and TIA [23]. In our study, the BFV of ipsilateral MCA was the lowest in the OA group. The possible reason for these inconsistent results may be the different inclusion and grouping criteria. Besides AcoA and PcoA, there may be other collaterals (e.g. leptomeningeal artery) which have little effect on cerebral perfusion. Cheng et al. found cerebral perfusion to be significantly impaired in patients with collateral circulation via secondary collaterals, which was consistent with our findings [24].

### Relationship between frontal lobe cognitive function and collateral circulation

Some studies have proposed that cerebral BFV can reflect the brain perfusion as well as cognitive dysfunctions [25]. Wang et al. reported significant CBF reduction in patients with anterior circulation TIA who had vascular cognitive impairment [26]. Patients with carotid atherosclerosis showed lower scores in verbal fluency test even when the atherosclerosis were subclinical [27], and verbal fluency was improved after carotid artery stenting or carotid endarterectomy for patients with carotid artery stenosis [28]. These findings indicated that CBF was associated with verbal fluency performance. In the present study, decreased performance of Word Fluency Test in the PcoA and OA groups indicated their impaired sematic memory and language ability. The undifferentiated results between the AcoA and control group implied a potential for preseverd language function among AcoA collateral patients. It has been reported that patients with greater carotid artery intima-media thickness had worse TMT performance [29], which is consistent with our findings. Digit Span is a measure of working memory and is not associated with emotional lability [30]. The worse results of TMT and Digit Span in patients with ICA stenosis indicated impaired executive function and working memory included in frontal lobe cognitive function, and the type of collaterals had no significant impact on these subfunctions.

In SCWT, significant difference was only found in RT and AR of SCWT C and SIE, suggesting a higher sensitivity for detection of selective attention in patients with severe ICA stenosis. In SCWT C, subjects needed to suppress the habitual response to the meaning of the words and shift their attention to its incongruent color. The incongruent task in Stroop requires response inhibition, which may be linked with anterior cingulate gyrus [21]. Better performance in SCWT requires more blood flow in frontal lobe [31], and worse performance in patients with ICA stenosis may be explained due to the altered blood flow. Carter et al. hypothesized that the poor performance in SCWT may be associated with the anterior cingulate gyrus dysfunction as evaluated by positron emission tomography [32]. Moreover, Benabarre et al. found the SCWT score to be well correlated with the CBF in striatal, temporo-medial, and parietal cortices [33]. Taylor et al. found the activation of left inferior frontal gyrus on positron emission tomography to reflect the Stroop processing [34]. All of the regions mentioned can be affected by reduced blood supply of ICA. Furthermore, the dorsal anterior cingulate cortex receives top-down information from the dorsolateral prefrontal cortex and bottom-up information from the left caudate nucleus [35]. Hypoperfusion may disrupt the interaction of these grey-matter areas, leading to poor performance in SCWT. AcoA has been verified to supply the frontal lobe in patients with ICA occlusion [36]. We also noted patients with collaterals via AcoA exhibited a better performance in SCWT than the patients in other groups. Of note, RT_SCWT C_ and RT for SIE in AcoA group were longer than those of other groups. He et al. found the Chinese language functional areas were located in both frontal and temporal lobes [37]. Therefore, patients in AcoA group may suffer less impairment in language function because of the well-preserved anterior circulation. The normal results of the AcoA group in Word Fluency Test also demonstrated inconsistency. In SCWT, they could be easier to be interfered by the incongruent meaning of the words, and they might spend more time in judging the color of the words. However, there was no difference in AR for SIE only between the AcoA group and controls. We speculate the patients in AcoA group had relatively intact language function manifesting as normal AR at a cost of prolonged RT. Both RT and AR were increased in PcoA and OA groups, indicating that collaterals via PcoA or OA to be associated with a relatively severe impairment of selective attention. Additionally, AR_SCWT C_ in the OA group was significantly lower than in normal controls, suggesting the patients in the OA group had more severe selective attention impairment. Reinhard et al. proposed that the cerebral blood autoregulation in patients with collaterals via secondary pathways was worse than that in patients with collaterals via primary pathways [38], and cerebral autoregulation to be crucial for preserving the cognitive function, which may help explain our findings. In general, the frontal lobe cognitive function is impaired to various extents in patients with ICA stenosis. Being different from other frontal lobe cognitive assessment, SCWT, especially SCWT C and SIE reflected respective position and function for different collaterals more precisely.

### Relationship between selective attention and TCD parameters

We noted a negative correlation between Vd of MCA and RT for SIE in the OA group, indicating that BFV may be insensitive in reflecting selective attention because we only found statistically significant correlation between Vd and RT for SIE in the OA group. A possible reason is that the inconstant blood flow may be influenced by blood pressure, temperature and psychological state, and cerebral BFV may reflect selective attention only when cerebral perfusion is severely impaired.

The positive correlation between ipsilateral PI and SIE suggests that PI could be a potential indicator for evaluating selective attention impairment. In our patients with severe ICA stenosis, PI of ipsilateral MCA decreased in all patient groups. In previous studies, the relationship between PI and cognitive function remains controversial. Altmann et al. demonstrated that elevated PI was associated with impairment in several cognitive domains [39]. However, Shim et al. found that PI in patients with cognitive impairment did not differ from that in normal controls [40]. We speculate the impaired selective attention may be partly due to the abnormal resistance of cerebral vessels and low cerebral perfusion. Moreover, the more general correlation of PI and AR for SIE indicated AR may be a more valuable indicator in reflecting cognitive function in patients with ICA stenosis.

### Limitations

There are several limitations to the current study. First, the slight statistical difference in diabetes mellitus and hyperlipidemia between the AcoA group and the controls may be due to the limited sample size. However, the worse results of the PcoA and OA group showed the association of diabetes mellitus and hyperlipidemia with the impairment of frontal lobe selection may be nonexistent. Second, the present study was limited by the small sample size of other types of collaterals as only collateral circulation via AcoA, PcoA and OA was analyzed. Third, drugs (especially statins) may be confounding factor on vasomotor reactivity [41]. In the future, we will enlarge the sample size via initiating a multi-center study and take the drug-related confounding factors into account.

## Conclusions

To our knowledge, this study is the first to analyze the influence of different collaterals on CBF and frontal lobe cognitive function in patients with severe ICA stenosis. The frontal lobe cognition function is impaired to different extents according to the type of collaterals. Most notably, selective attention impairment is correlated with the type of collaterals. Collateral circulation via AcoA is associated with a relatively intact selective attention, and collateral circulation via OA is associated with a severe impairment of selective attention. Furthermore, PI may be a potential indicator for identifying selective attention dysfunction in patients with severe ICA stenosis.

## Data Availability

The datasets used and/or analysed during the current study are available from the corresponding author on reasonable request.

## Abbreviations

AcoA: Anterior communicating artery
ANOVA: Analysis of variance
AR: Accuracy rate
BFV: Blood flow velocity
CBF: Cerebral blood flow
DSA: Digital subtraction agiography
ICA: Internal carotid stenosis
MCA: Middle cerebral artery
MMSE: Mini-Mental State Examination
NASCET: North American Symptomatic Carotid Endarterectomy Trail
OA: Ophthmaltic artery
PcoA: Posterior communicating artery
PI: Pulsatility index
RT: Response time
SCWT: Stroop Color Word Test
SD: Standard deviation
SIE: Stroop interference effects
TCD: Transcranial Doppler
TIA: Transient ischemic attack
TMT: Trail-Making Test
Vd: Diastolic flow velocity
Vm: Mean flow velocity
Vs: Systolic flow velocity
